# Community Mental Health Practice in the United States: Past, Present and Future

**DOI:** 10.17650/2712-7672-2020-1-2-7-13

**Published:** 2020-12-04

**Authors:** Jay A. Hamm, Samuel Rutherford, Courtney N. Wiesepape, Paul N. Lysaker

**Affiliations:** Eskenazi Health, Midtown Community Mental Health; Purdue University, College of Pharmacy; University of Indianapolis; Indiana State University; Richard L. Roudebush, VA Medical Center; Indiana University School of Medicine

**Keywords:** community psychiatry, community mental health, schizophrenia, recovery, de-institutionalization, psychosis, амбулаторная психиатрия, амбулаторная психиатрическая помощь, шизофрения, восстановление, деинституционализация, психоз

## Abstract

Similar to trends in Europe, approaches to mental illness in colonial America and recorded in early United States history were commonly characterized by incarceration and the removal of individuals from communities. In the mid-20^th^ century, a major shift began in which treatment was offered in the community with the aim of encouraging individuals to rejoin their communities. In this paper, we will provide a brief history of community mental health services in the United States, and the forces which have influenced its development. We will explore the early antecedents of community-based approaches to care, and then detail certain factors that led to legislative, peer and clinical efforts to create Community Mental Health Centers. We will then provide an overview of current community mental health practices and evolving challenges through to the present day, including the development of services which remain focused on recovery as the ultimate goal.

## INTRODUCTION

The treatment of individuals with significant mental health needs within the United States has a long and complex history. Generally, one of the most significant aspects of that history was the development of community- based mental health services. Importantly, this included a transition from institution-based care to community- based care which took place in the mid-20^th^ century [1]. During this period, the United States experienced a dramatic decrease in the availability of institutional beds, alongside concomitant increases in the number of people seeking mental health services in outpatient, community settings. At a more in-depth level, these changes represented a shift more profound than the relocation of services; they were driven by changing ideas relating to the treatment provided by mental health services and the composition and character of those services. Any understanding of the development of community-based mental health services and more importantly, of its current state and future, thus requires consideration of the more complicated and underlying theoretical issues.

Accordingly, in this paper we will offer a brief history of community mental health in the United States, and the forces which have influenced its development to the present day. We will, firstly, explore the early antecedents of community-based approaches to care, before detailing certain major factors that led to legislative, peer and clinical efforts to create Community Mental Health Centers (CMHC) in the mid-20^th^ century. We will then provide an overview of current community mental health practices and their evolving challenges. Next, we will examine those challenges in light of deeper and more complex issues related to the meaning of care, and recovery as its ultimate goal, which are still being explored, as the field moves forward. We will, in particular, be emphasizing aspects of history and current practice, as they relate to the treatment of individuals with serious mental illness (SMI). As currently structured, community mental health includes a broad array of services for individuals with less severe difficulties, as well as child developmental issues and addiction services. Although these aspects are also essential elements of treatment within the United States, we suggest that many of the issues underlying shifts in treatment with regard to SMI, may largely be generalized in line with other aspects within the larger system.

## THE EMERGENCE OF COMMUNITY MENTAL HEALTH CARE

While the history of community care is generally considered to have begun in the mid-20^th^ century, much of its original focus, as well as initial efforts to implement it, have a circuitous history. Similar to trends in Europe, approaches to mental illness in colonial America and recorded in early United States history, were commonly characterized by incarceration and removal from communities. As madness came to be regarded as within the medical purview over time, the early pioneers of American medicine, such as Benjamin Rush, began implementing somatic interventions; early medical interventions included harmful practices such as bloodletting and blistering. Within this framework, treatment was aimed at attacking a 'disease', facilitating a cure and an eventual return home. In response, however, to increasingly inhumane conditions and harsh treatments, new approaches, including the Quakers' Moral Treatment approach, began to highlight the need for milder interventions, including a prescription of activities mirroring those that patients would experience in their own community [2]. Central to the Quakers' philosophy of care was openness to restorative treatments and a return to full participation in the community.

The view that temporary respite in an asylum could lead to full reintegration in the community, gradually eroded over the course of the 19^th^ century, as asylums and an ever-expanding array of experimental somatic interventions were developed. Trends towards longterm institutionalization with medical models directing conceptualizations and interventions, became increasingly prominent throughout the 19^th^ century arguably reaching maximum influence at the turn of the 20^th^ century, as Kraepelin's formulation of dementia praecox spread across the United States [3]. Within the paradigm of that era, there were no temporary or episodic aliments. Following Kraepelin's model, individuals were instead commonly viewed as experiencing a progressive medical illness, that required long-term institutionalization and custodial care, which were incapable of halting the deterioration of the illness [4]. This view, in combination with increasingly overpopulated institutions and eugenics laws advocating sterilization and dangerous interventions, such as early forms of shock therapy and psychosurgery, set the stage for rampant iatrogenic harm and abuse in mental institutions across the United States in the first half of the 20^th^ century. However, throughout this period of history, voices of reform raised concerns about the pessimistic prognosis levelled at severe mental illness and highlighted the harmful practices occurring in American mental institutions [5].

In the mid-20^th^ century, calls for the reform of institutional-based care were heeded, with several key pieces of legislation influencing national trends. In 1946, the National Mental Health Act was passed, creating funding for psychiatric education and research, and ultimately bringing about the creation of the National Institute of Mental Health in 1949. In 1963, the Community Mental Health Center Act and the Mental Retardation and Community Mental Health Centers Construction Act were both passed, prompting an increase in funding for the creation of centres in the community that provided a wide range of psychiatric services. Associated with the increased funding for community-based services was a decrease in funding for public mental hospitals and a dramatic decrease in the number of long-term hospital beds during the same time period. The creation of Medicaid and Medicare in 1965 furthhr facilitated the transition from large, public, psychiatric hospitals to the creation of community-based clinics, as well as nursing homes and intermediate care facilities, with a focus on treatment for individuals in the least restrictive environment. As emphasized by these initiatives, health was not only an attainable outcome, but could be achieved as a result of a connection with the community, and not as a precondition for that connection. Within eight years, a total of 398 community centres were in operation, approximately 0.18 per 100,000 people [6]. Over the next 40 years, tracked by the Substance Abuse and Mental Health Services Administration (SAMHSA), the number of community centres registered in the National Director of Mental Health Treatment Facilities in 2016 would reach 2,636 with a density of 0.73 per 100,000 people [7]. The staffing of these centres varied across time and region, but generally, community mental health centres could be expected to employ a mix of mental health professionals and paraprofessionals, including psychiatrists, psychologists, counsellors, occupational therapists, social workers, case managers, addictions counsellors and nursing staff, all of whom might be expected to have direct contact with patients.

## POST DE-INSTITUTIONALIZATION

Broadly, the legislative, clinical and advocacy efforts of the mid-20^th^ century within the United States were effective in achieving large-scale de-institutionalization of the overpopulated state facilities of the previous era. However, as more outpatient treatment centres were built across the country their efforts to help individuals with SMI regain their health and remain integrated in their communities were soon thwarted by several barriers. Of significance was the fact that the decades following the passage of the Community Mental Health Act were characterized by declining funding for outpatient centres, which, in light of the increased demand for mental health services, resulted in increasing levels of unmet mental health needs. The growth of mental health services has also not kept pace with the growth in population. From 2000-2017 the population of the United States grew by approximately 42 million. During that time period, the rate of psychologists and psychiatrists per 100,000 people, remained largely the same; between 36.55 and 33.18, and 7.54 and 7.75, respectively [8].

Efforts were made to reform the American healthcare system by increasing third-party reimbursement and streamlining service utilization. In the 1980s, this was reflected in the emergence of behavioural health-managed care. Managed care involved private companies that dictated service authorization, utilization, claims processing and interagency coordination, with the intention of promoting improved efficiency and effectiveness of mental health services. With limited public funding to support CMHC, agencies became increasingly reliant on these third-party reimbursements, necessitating interface with managed care entities, as well as demonstrating parallel trends within Medicare and Medicaid. This led to organizations structuring service delivery programmes in order to receive reimbursement from a patient's insurance plan.

While the emergence of managed care in the United States allowed for the survival and expansion of mental health services, in some ways, it probably resulted in a shift away from addressing the needs of individuals with SMI, many of whom were unlikely to have access to insurance or third party reimbursement. The most obvious negative impact of this was that individuals treated within their community were often unable to sustain satisfactory community participation, ultimately resulting in them being incarcerated. In other words, without adequate support, the attempt to help individuals move from asylums into the community led to larger numbers of these individuals challenging societal norms, resulting in legal convictions and boosting the number of state and federal prisoners with histories of significant mental health disorders [9] [10]. These observations suggest that the path to de-institutionalization was reversed for some [11] [12]. This, along with the more significant issues of underfunding, may also have contributed to a growing pessimism as to whether individuals with SMI could actually become well and fully re-join their communities; views that can be linked with long traditions of paternalism, coercion and control [13].

The changing financial and political landscapes, in combination with increased treatment of SMI in prisons rather than the community, led to a cultural shift as to the meaning of wellness, in relation to SMI. Notably, in 2003, the New Freedom Commission on Mental Health was commissioned to study the status of the mental health delivery system and provide recommendations for the vision of mental health in the 21^st^ century. The report issued by this commission in 2004 embraced the contributions of the recovery movement within the United States and established at least three expectations [14]. Firstly, it stated unequivocally that recovery was the expected outcome for mental health and substance abuse treatment. Secondly, it defined recovery as patient centred and as a journey that involved hope, autonomy and self-determination. Finally, the commission called for the development of recovery-oriented mental health services. While this report did not solve the problem of prisons emerging as long-term treatment facilities for SMI, it did spur on the development of new forms of recovery-oriented services, including peer counselling [15] and other interventions focused on psychosocial outcomes.[16] [17] [18]

## CURRENT STATUS

Currently, in the United States, federal governmental regulations determine the monetary value distributed to individual states, as well as determining how monies can be spent. The majority of federal dollars for mental health services are distributed by means of Mental Health Block Grants via SAMHSA. Since its inception in 1992, SAMHSA has contributed varying amounts of money in the form of state-specific block grants to support and grow community mental health and substance abuse treatmeet centres. From an initial distribution of 1.69 billion dollars in 1992, SAMHSA has seen a gross increase in yearly distributions, ranging from a low of 3.12 billion in 2007, to a high of 4.2 billion in 2017 [19]. Additional federal funding sources come in the form of Veterans Administration Benefits and Medicare/Medicaid expenditures. Currently, there is considerable regional variability in the availability of community-based mental care, due to differing levels of local funding, the specifics of state Medicaid and the availability of providers.

As previously noted, by 2017 the United States had a total of 2,381 CMHC, with a density of 0.73 per 100,000 people. The services offered are provided by a broad range of professionals, including psychiatrists, psychologists, counsellors, occupational therapists, social workers, case managers, addictions counsellors and nurses. Services are provided in group, family and individual formats and are expected to be individually tailored to meet the unique needs of any given patient. General services, commonly available, include medication management, case management, and group, family and individual therapies. These types of services should be capable of responding to the full range of psychosocial needs and therefore, vary significantly from site to site, with common services including supportive, psychoeducational, vocational, social, addiction, educational and activity-based interventions. These interventions can be delivered within the physical space of the CHMC or in the home or community of the patient. Additionally, there are state-wide disparities in CMHC densities; California, the most populous state, has 0.22 per 100,000 people, while Wyoming, the least populous state, has 4.15 per 100,000 people. These numbers are dwarfed by the 7,482 for-profit mental health agencies in the United States [7]. The majority of mental health services are provided by agencies that charge insurance premiums or require self-payment at the time of service. Of those seeking mental health treatment, 42% see cost and lack of insurance coverage as the greatest obstacle to accessing services, with 25% stating that they are faced with the dilemma of obtaining mental health services or paying for daily necessities [20].

Licensed professionals practicing across these settings include psychiatrists and psychologists, commonly functioning as the providers of records, while much of the direct service is conducted by social workers, mental health therapists or addictions counsellors, with either a master's or a bachelor's degree. Healthcare workers from a range of other disciplines, such as nurses, occupational therapists, pharmacists, dietitians and primary care physicians are also found in certain community mental health settings. The expectation for community mental health care involves a continuum of services, not limited to psychiatric medicine, nursing intervention, supported housing and supported employment. Additionally, a host of psychosocial services, consisting of individual and group psychotherapies, skills- based psychosocial rehabilitation, case management and a range of peer services, including individual support, self-help approaches and peer-led clubhouse services are also provided. These services are delivered across a range of settings, including standard outpatient health clinics, in patient's homes or in the community (e.g., grocery stores, coffee shops, government offices, homeless outreach premises, job settings, etc.). Certain community mental health settings are directly integrated with primary care medicine, while others must link their services with other sources of primary care in their communities. Following the national economic recession in the United States, healthcare-related jobs increased between 2008 and 2009, while all other industries saw reduced growth; the total number of health care jobs created between 2007-2013 had a value of approximately 1.85 million. Most new jobs in healthcare were positions that required less formal education, particularly jobs with high rates of turnover. The dramatic increase in the number of positions was due, to a great extent, to the Affordable Care Act [21].

## THE EVOLUTION AND FUTURE OF COMMUNITY MENTAL HEALTH SERVICES IN THE UNITED STATES

As discussed at the outset of this report, the community mental health movement in the United States, particularly with regard to the needs of adults with SMI, has, for nearly 70 years, been driven by a vision of treatment that promotes recovery and integration within one's community. As this has unfolded, many social and economic issues have occurred, leading to a rocky progression; prisons have become the new asylums for some, while the professional work force has not grown to meet emergent needs. Looking to the future, we certainly do not see simple solutions to these problems. We do, however, see potential developments which may help offset some of these challenges, as well as counter movements which could resurrect even more intransigent barriers to recovery.

The most significant developments which we see affecting the future of community mental health in the United States are the emerging, nuanced views as to what recovery represents, linked to the way in which services need to be developed and implemented to support recovery. In our view, studies of the experience and perspective of the individual with SMI, including qualitative and quantitative studies, as well as user-led participatory research [22], have revealed that recovery means attaining a satisfying and meaningful life, with healthy and sustaining, interdependent connections with one's community. Recovery does not constitute the absence of a disease, the development of a skill or anything else that could be considered as happening within one person in isolation [23] [24]. Instead, recovery is a return to full participation in the larger, human community [25]. Interventions are thus needed that support individuals with SMI in making meaningful sense of their own perception of mental health and its related life challenges, prior to facilitating their decision making with regard to responding to these challenges [26].

Importantly, this view of recovery precludes the fact that just one treatment could promote recovery for all. Instead, integrative approaches are needed that go beyond generic support and can be customized to address individual needs and processes of selfdirection. Such integrative treatment frameworks would need to be flexible so that service providers could consider and respond to the individual, using a holistic approach. Similarly, these frameworks would have to move from a didactic or paternalistic model to a fully consultative one. These new models would also need to move beyond the implementation curriculum, which focus on singular problems or skills; instead they would have to be able to flexibly help individuals respond to the myriad of psychiatric, social and psychological problems, which may emerge fluidly during the course of recovery. Examples of recent work, inspired by these newly defined needs, include Metacognitive Reflection and Insight Therapy (MERIT) [24] [27] [28], Open dialogue [29], as well as clubhouse-based approaches [25].

It is important to note that the development of treatments that are able to address meaning in this way face stiff challenges in our current environment. There are competing ideas relating to recovery, which is still considered as a state defined by professionals, rather than an evolving condition experienced by those suffering from mental illness. Additionally, patient- centred treatmeets face resistance from conventional treatment approaches, as well as social hysteria, linked to the need to compartmentalize and marginalize those deemed dangerous or unwell in the eyes of the community. These counter trends, while understandable in some cases, threaten a regression to past methods of treatment which, although taking place in the community, do not facilitate community membership. Polarization within the psychiatric community may also be fuelling these negative trends. Traditional approaches to mental health, including models of schizophrenia as presented in the DSM 5 [30], neglect the concept of recovery, while other approaches, concerned with the issue of autonomy, call for the dismantling of structures within community mental health, which although flawed, have enabled meaningful recovery work to take place and seem necessary for the growth of future interventions.

## SUMMARY

The current community mental health system in the United States was initially developed in the mid-20^th^ century through resurrected values of restorative care from early efforts, including Moral Treatment. The guiding vision of recovery is that people experiencing mental illness should be offered services that help them live as full members of the community, seeking lives of meaning and value, with or without persistent symptoms and/or disability. In the decades following the creation of the original community mental health centres, reduced funding and increased demand has led to difficulties relating to access to care and a large number of people with mental illness finding themselves in the criminal justice system. Increased awareness of this difficulty, as well as the influence of the recovery movement, managed care practices and the more recent changes to the national healthcare system, are likely to continue to influence the evolving nature of available services in the community.

## Authors contribution

Jay A. Hamm: performed the literature review and developed the conceptual outline for the paper, worked on the first draft; Samuel Rutherford: performed the literature review and worked on the first draft; Courtney N. Wiesepape worked on the evolving drafts; Paul H. Lysaker: performed the literature review and developed the conceptual outline for the paper, worked on the first draft.

## Conflict of interest

The authors declare no conflict of interest.

## Funding

The authors declare that there was no funding for this work.
